# Aneurysm of the arch of the great saphenous vein: therapeutic challenge and review of the literature

**DOI:** 10.1590/1677-5449.202401072

**Published:** 2025-01-10

**Authors:** Badr El Kassimi, Abdelkarim Kharroubi

**Affiliations:** 1 Souss Massa University Hospital Center, Agadir, Morocco.

**Keywords:** aneurysm, great saphenous vein, saphenous vein junction, aneurisma, veia safena magna, junção da veia safena

## Abstract

Venous aneurysms are uncommon and can involve the entire venous system and occur at any age. The presence of these aneurysmal formations at the level of the saphenous vein junction is rarely reported, given the small number of cases described in the literature. We report the case of a 41-year-old patient with an aneurysm in the saphenofemoral junction of the right great saphenous vein, discovered incidentally during a consultation for varicose veins of the right lower limb.

## Resumo

Os aneurismas venosos são raros, podendo envolver todo o sistema venoso e ocorrer em qualquer idade. A presença dessas formações aneurismáticas no nível da junção da veia safena magna é raramente relatada, dado o pequeno número de casos descritos na literatura. Relatamos o caso de um paciente de 41 anos com um aneurisma na junção safenofemoral da veia safena magna direita, descoberto incidentalmente durante uma consulta para varizes do membro inferior direito.

## INTRODUCTION

Venous aneurysms are uncommon and can involve the entire venous system, occurring at any age. The most frequent location is in the lower limbs, most often in the popliteal vein. The presence of these aneurysmal formations in the saphenofemoral junction is rarely described in the literature, given the small number of cases reported, and they are generally asymptomatic and detected incidentally. Their origin is most often congenital, but they can also be acquired.

This study was approved by the Research Ethics Committee at the institution to which the authors are affiliated. The authors state that the manuscript is in accordance with the Helsinki Declaration and with local ethical guidelines. Informed consent was obtained.

### Part I – clinical situation

**Figure 1:**
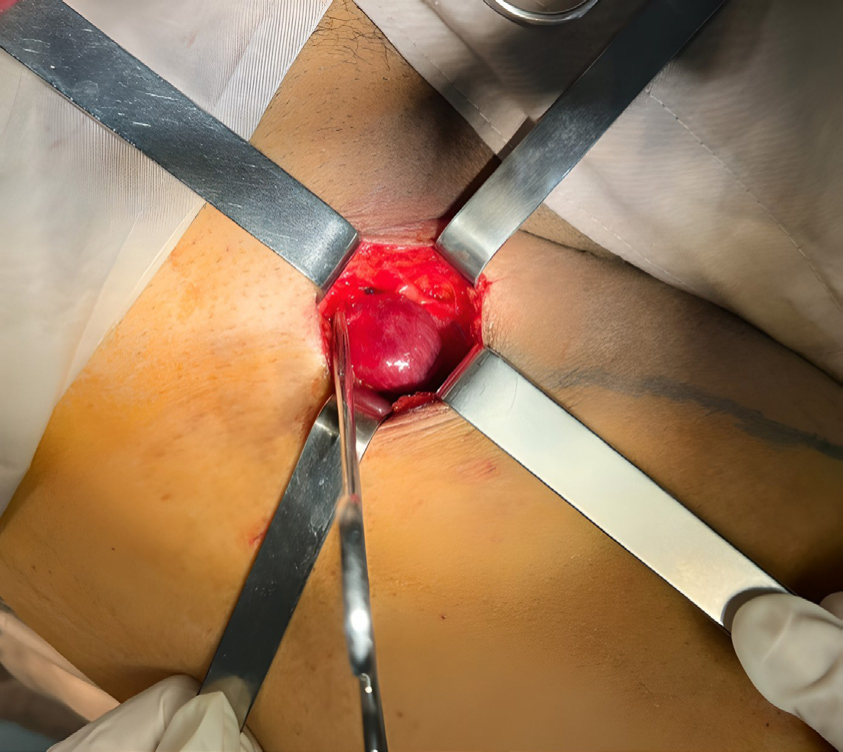
Venous Doppler ultrasound image of the right upper limb showing aneurysmal dilatation of the arch of the great saphenous vein.

This 41-year-old patient, with no previous pathological history of note, presented with painful venous dilatation of the right inner thigh, associated with right premalleolar ochre dermatitis. Clinical examination revealed a soft, non-throbbing, painless, right subinguinal swelling. Varicose veins were reticular and systematized along the course of the right great saphenous vein (GSV). Venous Doppler ultrasonography showed an aneurysmal dilatation of the initial portion of the GSV, measuring 25 mm (Figure 1). The GSV was dilated, incompetent at the level of the saphenofemoral junction and along its course, measuring 13 mm at the level of the saphenofemoral junction and 12 mm at the level of its trunk. The iliofemoral-popliteal venous axis was free, permeable, and compressible.

### Part II – what was done

**Figure 2:**
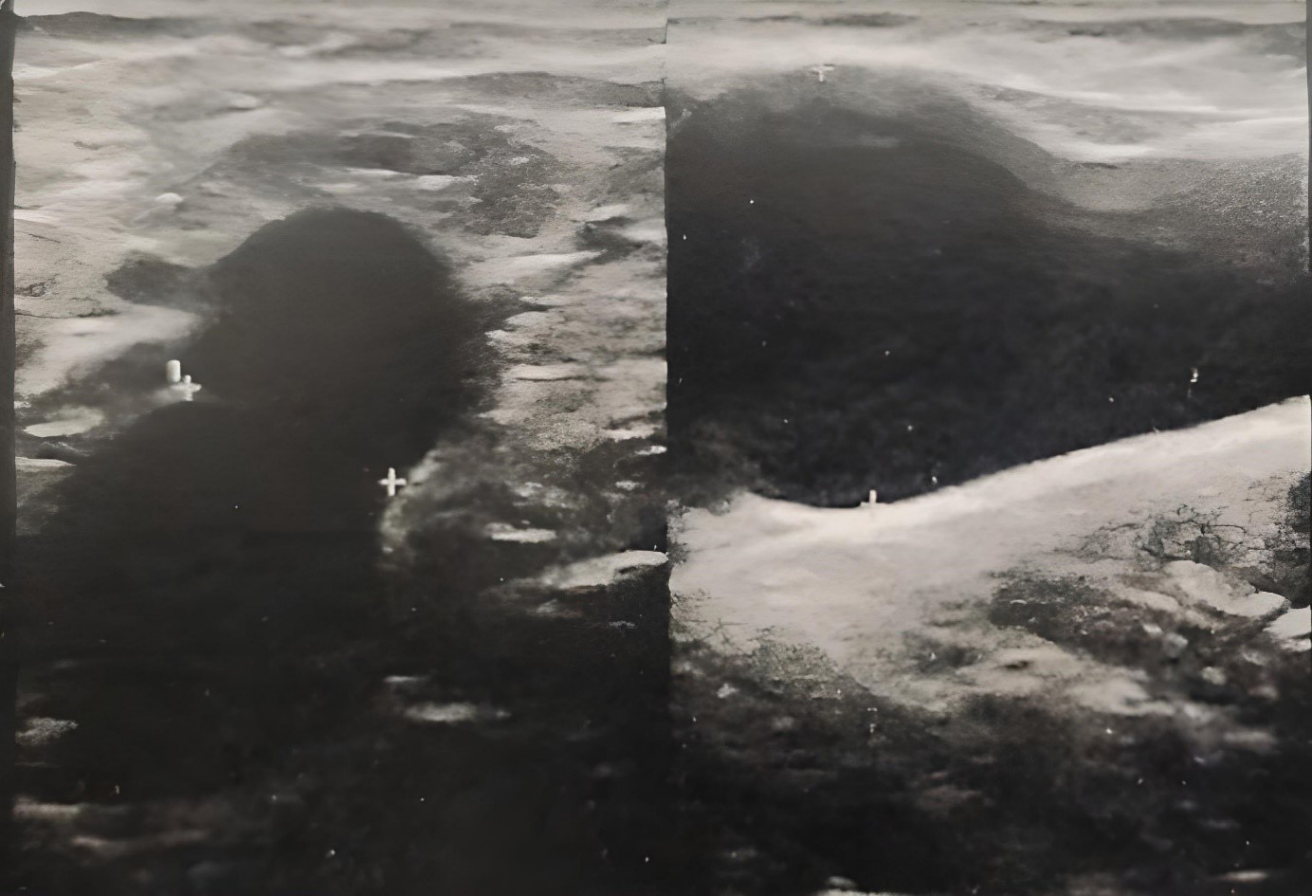
Intraoperative view of a saccular aneurysm of the arch of the right great saphenous vein.

The procedure involved resection of the aneurysm with crossectomy, suture of the femoral vein, and stripping of the right GSV. The contents of the aneurysm were thrombosis-free liquid blood and the wall of the vein itself was clean (Figure 2). The patient was put on preventive anticoagulants and prophylactic amoxicillin-based antibiotic therapy for 1 week, with a consultation appointment after 1 week.

Histological confirmation shows a thickened and weakened aneurysm wall with fibromuscular dystrophy.

Follow-up period: The patient was seen 15 days postoperatively with good clinical progress.

## DISCUSSION

Venous aneurysms are rare vascular anomalies, which can involve the entire venous system.^1^ They are generally asymptomatic and are discovered either by clinical examination, in the presence of a painful mass or a complication such as deep vein thrombosis or pulmonary embolism,^2^ or by paraclinical examination in patients with chronic venous insufficiency.

Pascarella et al. propose 4 types of saphenous vein aneurysm, depending on location. In type I, the aneurysm is located in the proximal third of the saphenous vein, not at the saphenofemoral junction. Type II venous aneurysms are located in the distal third of the thigh, in the diaphysis of the saphenous vein. Type III is the coexistence of types I and II in the same extremity. Type IV is an aneurysm of the small superficial saphenous vein.^3^

A histological study of the aneurysm wall by Horakova et al.^4^ highlighted a reduction in all layers of the venous wall, especially the media and adventitia, with disintegration of the bundles of smooth muscle in the media and a reduction in all layers of the wall. In other segments of the aneurysm wall, the structure shows disorganization of the smooth muscle cells in favor of fibromuscular dystrophy.

Aneurysms are more frequent in the superficial network of the limb, and in the presence of valvular incompetence, strong, cyclic reflux leads to ectasia or aneurysmal dilatation. The latter is most often saccular and is often a varicose degeneration all along the saphenous network, including the saphenofemoral junction.^5^

Venous Doppler ultrasound allows us to highlight the aneurysm, its location and measurements, making it the preferred non-invasive test for diagnosing deep vein thrombosis and venous aneurysms.^6^ This ultrasound technology provides high-quality images that accurately define the anatomy and pathological state of the sub-inguinal venous system.^7^

The risk of thromboembolism is real in deep locations, notably popliteal, but virtually non-existent in superficial locations.^8^ However, cases of pulmonary embolism resulting from superficial venous aneurysms have been reported.^9^

According to de Miranda et al.,^10^ the surgical techniques proposed for venous aneurysms of the limbs include excision and simple ligation; excision and interposition of vein or prosthesis; tangential excision with lateral venorrhaphy; and total resection and placement of venous patch. For the superficial network there is no need for reconstruction. In our case, the aneurysm was resected with crossectomy and stripping.

The subinguinal location of aneurysms of the saphenopopliteal junction is a source of confusion with crural hernia. Merghit et al.^11^ have reported a case of differential diagnosis in the crural region with aneurysm when it is clinically manifest.

## CONCLUSION

Most venous aneurysms are located in the saphenous trunks, but their location in the saphenofemoral junction is so uncommon that they should be considered in the presence of any compressible swelling at the root of the thigh. venous Doppler ultrasound makes diagnosis easy. Surgical management is essential, involving simple resection and ligation.
